# Selection of Multiarmed Spiral Waves in a Regular Network of Neurons

**DOI:** 10.1371/journal.pone.0069251

**Published:** 2013-07-29

**Authors:** Bolin Hu, Jun Ma, Jun Tang

**Affiliations:** 1 Department of Physics, Lanzhou University of Technology, Lanzhou, China; 2 College of Science, China University of Mining and Technology, Xuzhou, China; University of Maribor, Slovenia

## Abstract

Formation and selection of multiarmed spiral wave due to spontaneous symmetry breaking are investigated in a regular network of Hodgkin-Huxley neuron by changing the excitability and imposing spatial forcing currents on the neurons in the network. The arm number of the multiarmed spiral wave is dependent on the distribution of spatial forcing currents and excitability diversity in the network, and the selection criterion for supporting multiarmed spiral waves is discussed. A broken spiral segment is measured by a short polygonal line connected by three adjacent points (controlled nodes), and a double-spiral wave can be developed from the spiral segment. Multiarmed spiral wave is formed when a group of double-spiral waves rotate in the same direction in the network. In the numerical studies, a group of controlled nodes are selected and spatial forcing currents are imposed on these nodes, and our results show that *l*-arm stable spiral wave (*l* = 2, 3, 4,...8) can be induced to occupy the network completely. It is also confirmed that low excitability is critical to induce multiarmed spiral waves while high excitability is important to propagate the multiarmed spiral wave outside so that distinct multiarmed spiral wave can occupy the network completely. Our results confirm that symmetry breaking of target wave in the media accounts for emergence of multiarmed spiral wave, which can be developed from a group of spiral waves with single arm under appropriate condition, thus the potential formation mechanism of multiarmed spiral wave in the media is explained.

## Introduction

Spiral waves are ubiquitous in various spatiotemporal systems such as physical, chemical and biological systems [1]–[6]. An emergence of spiral waves in excitable media gives a fascinating example of self-organization in complex system, spiral wave solutions are approached when a well-stirred system with diffusion or coupling undergoes a Hopf bifurcation. The tip of a stable rotating free spiral wave moves around a circular core, which plays a role of an effective obstacle. The dynamics of spiral waves is dependent on the movement of its tip(singularity in the core), which is regarded as a topological defect. In a chemical reaction-diffusion system, the colors of products often change with concentration of products and reactants far from equilibrium state. In a biological system, the fluctuation of membrane potentials in neurons or myocardial cell can show star-bright snapshots with different profiles. In a numerical or theoretical way, a reaction-diffusion system [7], [8] is often used to simulate the formation and selection of spiral wave with a single arm [9], multiarmed spiral waves [10]–[20] with *l*-arm (*l* = 2, 3,4...), superspiral wave [21], [22]. The selection and suppression of spiral waves in excitable and/or oscillatory media have been investigated extensively due to its potential applications in preventing the occurrence of ventricular fibrillation [23]–[30]. On the other hand, *Huang et al.* [31], [32] experimentally observed the emergence of spiral waves in disinhibited mammalian neocortex. *Schiff et al.* [33] investigated the dynamical evolution of spiral wave in mammalian neocortex. It gives us a practical tool for measuring spatiotemporal patterns of population neuronal activity in the neocortex based on the voltage-sensitive dyes and fast optical imaging techniques. As a result, the dynamics of spiral waves in the coupled oscillators or network of neurons [34]–[41] has been investigated, and these interesting insights are useful to understand the fundamental properties of the selection of spatiotemporal patterns in network.

Information coding [42]and various of signals communication among neurons are much dependent on the collective behavior of a large number of neurons in the neuronal system. A quantitative study of electrically active cells started from nerve conduction in the squid giant axon [43], [44], then a Hodgkin-Huxley(HH) model with four variables [45], [46] is used to model the membrane potential and ion conductances at a fixed point in the axon, and the improved cable model with a diffusion term [47] is used to measure the propagation of an action potential along the fiber and is often compared with experimental data. The electric activities of neurons are associated to the conductance, the external electric forcing [48], [49], noise and other bifurcation parameters [50]–[54]. Particularly, an overview [55] is presented of the mechanisms through which noise induces, enhances, and sustains ordered behavior in passive and active nonlinear media, and different spatiotemporal phenomena are described resulting from these effects of noise on the media. Consequently, distinct regularity and ordered wave could be observed in the network of neurons(small-world [56] or regular connection type) due to stochastic and/or coherence resonance [50], [57]–[59]. Based on some experimental results [31]–[33], selection, formation and transition of spiral waves in the network were ever investigated in a numerical way, and the formation mechanism of spiral wave in the network was discussed [41]. It is confirmed that spiral waves can play a positive role in breaking through quiescent areas of the brain as a pacemaker, and coherence resonance-like behaviors occur. Spiral wave with a single arm is usual and observed in experimental and numerical ways. However, spiral wave with several arms (multiarmed or multi-arm spiral wave) is infrequent. Multiarmed spiral waves emerge in some biological systems and experimental systems [16], [17], [60]–[62]. It is found that multiarmed spiral waves are not spontaneous in chemical systems but can emerge under appropriate experimental conditions. Multiarmed spiral wave could be developed from a group of broken waves only when the distances among the tips are less than a wave lengthen, and the these spiral segments rotate in the same direction [13]. Multiarmed spiral waves are unstable in the media with high excitability but these waves could be stabilized by imposing external field on the media [63].

It is argued that neurons should be coupled in a small-world connection type, which can be measured by a local regular connection conjugated long-range connection with certain probability. In the case of pattern formation, a local regular connection is beneficial for survival of spiral wave whiles the long-range connection often deforms the ordered spiral wave, for example, spiral wave exists only when the probability of long-range connection *p* decreases to a certain threshold [37]. As a result, a regular network can describe the main collective properties of neurons in a small-world network when the probability of long-range connection is very small(e.g. 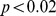). As reported in Ref. [41], spiral wave with single arm could be developed in the network of neurons when ordered wave is broken by artificial defects or local blocking in ion channels. Furthermore, the emergence of stable target wave in the network of neurons is also discussed by imposing spatial forcing currents on the network of neurons [64], which a single target wave could be induced in the network when the external forcing currents on two different areas of the network are selected with two constants with diversity. Indeed, diversity or variability in parameter can induce spatial coherence and collective regularity in coupled media. For example, Gosak [65] investigated the role of cellular variability on the occurrence of Ca 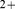 wave propagation in a net of diffusively coupled cells, and confirmed a resonance-like response due to the cellular variability by analyzing the spatial profile via the autocorrelation function. Glatt et al. [66] studied the pattern formation in subexcitable net of FitzHugh-Nagumo elements with parameter variability (diversity) being considered, an intermediate variability strength induced similar spatiotemporal stochastic resonance generated by additive noise in subexcitable media and transition induced by variability in coupling intensity is also observed. Surely, a local periodic forcing is also effective to generate a target-like wave in the network as well. More interestingly, it is worthy of detecting the collective electric behaviors of neurons in network when spatial forcing currents are imposed on the network, and the collision dynamics of a group of target waves and the development of target waves due to collision could be more attractive. In this paper, it will investigate the selection of multiarmed spiral waves in the regular network of Hodgkin-Huxley neurons with with a nearest-neighbor connection type. In our numerical studies, spatial forcing currents are imposed several groups of nodes to generate a group of double-spiral waves under ion channel blocking with certain degree. The formation of multiarmed spiral waves could be developed from a group of stretched double-spiral waves, and 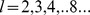-arms spiral wave could be induced in the network under excitability with diversity, and the selection criterion for multiarmed spiral waves is discussed.

## Model and Scheme

A regular network of neurons is designed by placing neurons on the nodes uniformly in a two-dimensional square array, the dynamics of each node is described by a Hodgkin-Huxley (HH) neuron model and the neurons are coupled with gap junction type or voltage coupling. The dynamics of membrane potentials of coupled HH neurons with nearest-neighbor connection is described as follows:
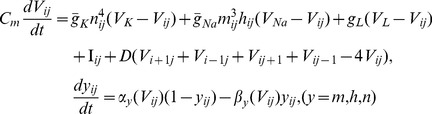
(1)

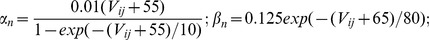


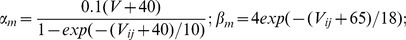


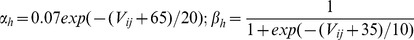
(2)

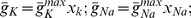
(3)where the variable 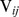, 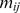, 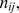 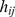, 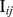 denotes the membrane potential, variable for gate channel, and intensity of external injected current on the neuron in the node (*i*, *j*), respectively. And the parameter 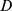 is the coupling intensity, 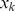 defines the ratio of active potassium channel number to the total potassium channel number 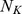, and 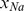 gives the ratio of active sodium channel number to the total sodium channel number 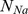. A higher ratio 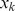
(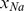) represents a lower degree of channel poisoning and a large number of ion channels are working [53]. The excitable media is isotropical and the capacitance of the membrane is 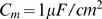. The maximal conductance of potassium is 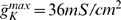, the maximal conductance of sodium is 
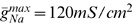
, the conductance of leakage current is 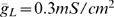. The physiological parameters for reversal potentials are selected as 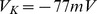, 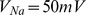, 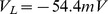. The spatial forcing current 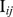 on neurons in different nodes initiates diversity in excitability of neurons in the network, for simplicity, intensity of the forcing current on some sampled nodes is marked as 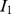, and the other neurons in the rest nodes of the network are imposed forcing current as 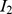.

## Numerical Results and Discussion

**Figure 1:**
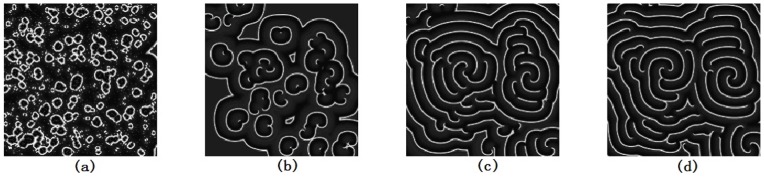
The evolution of spatiotemporal patterns in the network is plotted under spatial forcing current. The spatial forcing currents are selected as 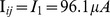 at 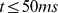; 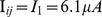 at 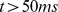, and are imposed on 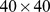 nodes with stochastic distribution in the network, for *t* = 50 ms(a), *t* = 100 ms(b), *t* = 300 ms(c), *t* = 500 ms(d). The snapshots illustrate the distribution for membrane potentials of neurons in gray scale, the coupling intensity 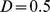, parameter ration 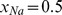 at 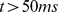; 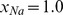 at 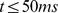, the forcing currents on the rest nodes are 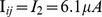.

In this section, 40000 HH neurons are placed in a two-dimensional square array to construct a regular network with 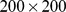 nodes with a nearest-neighbor connection. The initial states for all neurons in the network are selected as 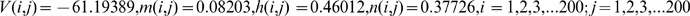. The time step 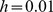, the coupling intensity 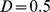, 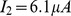 and no-flux boundary condition is used. It is confirmed that target-like wave could be developed when the spatial forcing currents with diversity(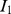, 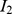) are imposed on the network, and spiral waves emerge when the target waves are broken by defects. In fact, the developed target wave or ordered wave could also be broken to form spiral waves when the excitability in a local area is changed. Inspired by the results [13] that multiarmed spiral waves can emerge in low excitability media by changing parameters in the model randomly. It is also interesting to investigate the selection of multiarmed spiral waves in the network in another feasible way. At first, 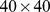 nodes are selected randomly and imposed the same forcing current 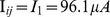, and the other nodes are imposed forcing current 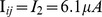; the forcing current on the randomly selected nodes is decreased to 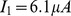 at 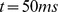, and then the parameter ratio 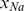 is switched from 1 to 0.5 at 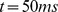 so that the excitability and conductance of sodium could be decreased, and a group of spiral segments(or double-spiral waves) are generated. As a result, the developed target wave is broken into many spiral segments and double spirals, then multiarmed spiral waves can be developed after frequent collision among these broken waves, and the results are shown in Fig. 1.

**Figure 2:**
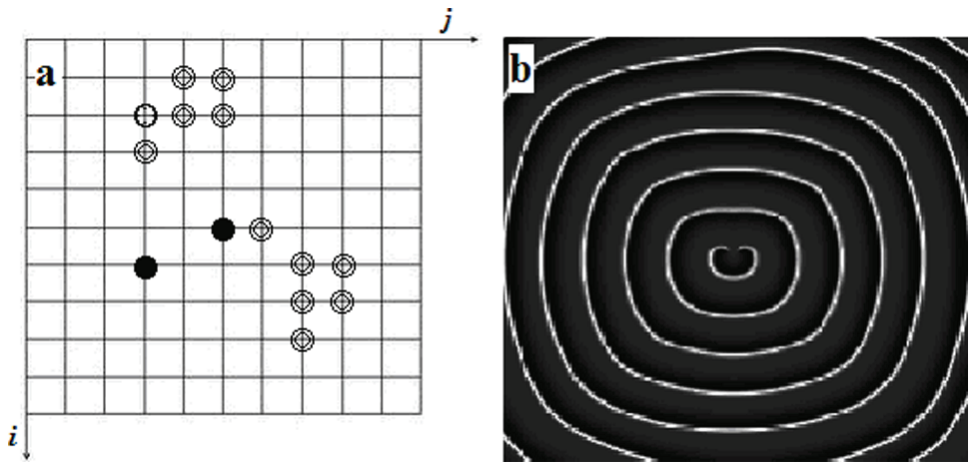
The diagrammatic sketch (a) for spatial forcing currents on the network. Spatial forcing currents 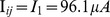 are imposed on the nodes(two solid black spots and one of the circles) in the network, the developed pattern for *t* = 200 ms. The snapshot(b) illustrates the distribution for membrane potentials of neurons in gray scale, the coupling intensity 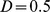, the forcing currents on the rest nodes are 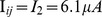.

The results in Fig. 1a show that many target-like waves could be induced in a local area in the network under spatial forcing currents with stochastic distribution, furthermore, these local target waves are broken to form spiral waves when the conductance of Sodium is decreased. In fact, the wavefront of the target wave propagates outside without persistence when the diversity between forcing currents 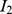 and 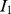 is removed, a sharp shift in the conductance of sodium make the wavefront break and thus spiral waves emerge in the network. Extensive numerical results confirm that no distinct multiarmed spiral wave can occupy the network completely but a group of spiral waves coexist in the network. Clearly, a spiral segment could be approached by a polygonal line connected with three points, a double-spiral wave emerges when the spiral segment is elongated. Multiarmed spiral wave could be developed when a group of double-spiral waves rotate in the same direction synchronously. Then we investigate this problem when the number of nodes injected by forcing current 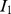 is decreased to 3, and the results are shown in Fig. 2.

**Figure 3:**
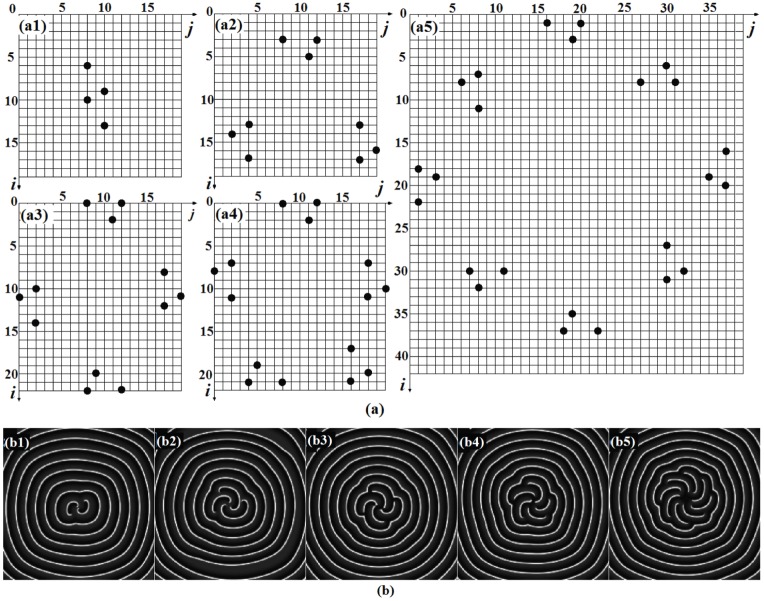
The diagrammatic sketch (a) for spatial forcing currents on controlled nodes in the network. Spatial forcing current 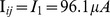 at 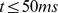 (and 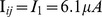 at 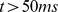) are imposed on the nodes(solid black spots) in the network. (a1) for 2-arm spiral wave, (a2) for 3-arm spiral wave, (a4) for 4-arm spiral wave, (a5) for 8-arm spiral wave. The developed pattern for *t* = 200 ms(b)(enhanced online). The snapshots(two-arm, three arm, four-arm, five-arm, eight-arm spiral waves) illustrate the distribution for membrane potentials of neurons in gray scale, the coupling intensity 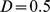, the forcing currents on the rest nodes are 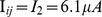.

The results in Fig. 2 show that a double-spiral is surrounded by a powerful target wave, and extensive numerical results confirm that the double-spiral wave does coexist with the target wave stably. The potential mechanism is that ambient target wave is induced when the three nodes are imposed the same forcing currents 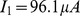, and the target waves generated from the three nodes began to interact with each other, and a double-spiral is formed due to breakup of the target waves in a local area when the forcing current on the three nodes are removed. Furthermore, we change the number and position of nodes injected forcing currents 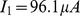, it is found that spiral waves with different arm numbers can be induced in the network and results are shown in Fig. 3(enhanced online).

**Figure 4:**
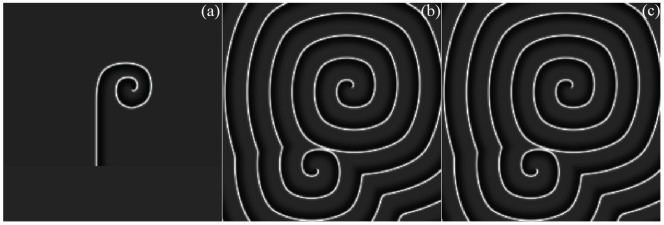
The development of double-spiral wave is plotted in the network. For 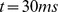 (a), 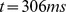 (b), 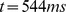 (c). Forcing currents on all nodes are 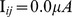, the coupling intensity 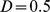, time step 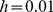, and no-flux boundary condition is used. The Fig. 4a is induced by selecting appropriate initial vales with a transient period about 30 ms then the arm of the spiral wave is cut off at 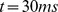. The initial values are selected as follows, 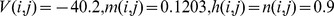 at 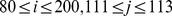; 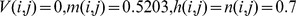 at 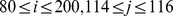; 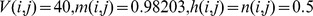 at 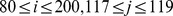; otherwise, 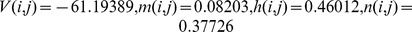 for the rest nodes.

The results in Fig. 3(enhanced online) show that 2-arm, 3-arm, 4-arm, 5-arm, 8-arm spiral waves could be developed in a local area of the network, and target wave still emerges outside of these spiral wave with *l*-arms(*l* = 2, 3, 4,...). The formation of *l*-arms spiral wave is dependent on the selection of number and position of nodes injected by 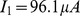. It is found that the position of nodes injected by 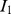 should be symmetric so that the spiral wave (in a local area)generated from each group of the three nodes could be symmetric in space completely. Extensive numerical results confirm that the 2-arm spiral wave could be developed to occupy the network completely with certain transient period, while other multiarmed spiral waves just are suppressed and coexist with the target wave outside and these multiarmed spiral waves (*l* = 3, 4,5...) will degenerate to spiral waves with fewer number of arms. That is to say, the multiarmed spiral waves(*l* = 3, 4,5...) are not stable but transient. The potential cause could be that the excitability of neurons close to the tip of spiral waves are high, some tips of the spiral waves attract other tips of the spiral wave to form a stable spiral wave thus the number of arms for spiral wave is decreased until stable spiral waves are formed in the network. Therefore, it could be practical to induce stable multiarmed spiral wave by decreasing the excitability of neurons in the network. And it is also important to develop these multiarmed spiral waves to occupy the network completely. According to the results in Fig. 3(enhanced online), multiarmed spiral waves are composed of several double-spiral waves. At first, we discuss the development of double-spiral wave in the network, and the results are shown in Fig. 4.

**Figure 5:**
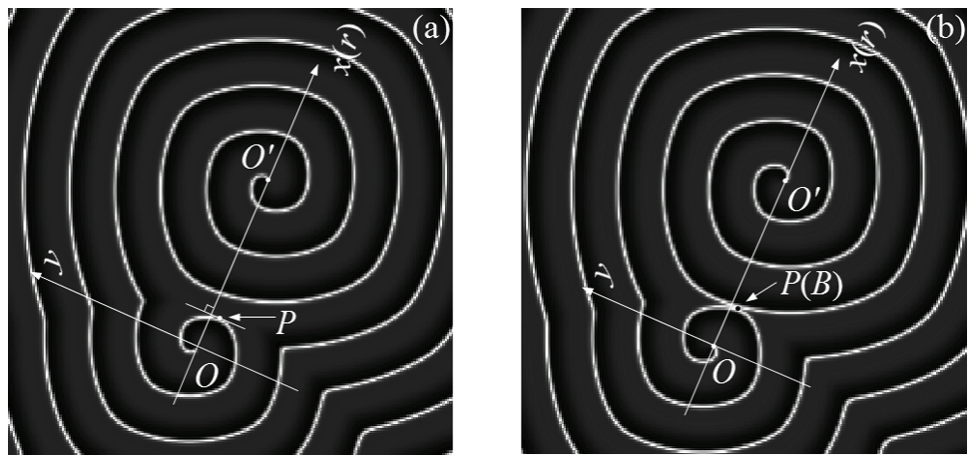
The diagrammatic sketch for the coordinate for the double-spiral wave in the network. In the case of polar coordinate, the connection line for point 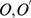 marks the polar axis, the point 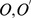 is regarded as the center for the tips of the double-spiral wave, respectively. Where 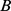 represents the intersection point for the wavefronts of the double-spirals, and 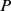 denotes the point of tangency on the wavefront of the outboard spiral wave and the corresponding tangent line is perpendicular to the polar axis.

The results in Fig. 4 confirm that a stable double-spiral wave could be developed but fails to grow up completely. In fact, in the isotropous network, the double-spiral wave rotates with the identical angular frequency as the target wave thus the two states coexist in the network of neurons. This result could be checked by analyzing the time series for the sampled membrane potentials of neurons in the network based on fast fourier transform (FFT). It is important to discuss the critical criterion for generating a growing-up spiral wave, a coordinate is builded in Fig. 4b(or Fig. 4c) and the origin coordinate is fixed at the center of the outboard spiral wave, and the diagrammatic sketch is shown in Fig. 5.

According to the results in Fig. 4, the double-spiral wave keeps alive, but it fails to stretch to the border of the isotropous network, which the dynamics for the nodes is identical. The coordinate as shown in Fig. 5 gives some clues to detect the critical criterion for supporting double-spiral wave with bigger radius of curvature, which the rotating spiral wave can occupy more nodes in the network. As a result, the dynamics of a stable rotating spiral wave could be approached by using a Archimedes helix in a polar coordinate as follows
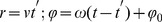
(4)where 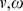 denotes the normal velocity and angular frequency, respectively. The time 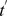 is the transient period to form a stable rotating spiral wave, 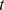 is independent of 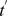, 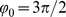 is the initial angle for tangent line on tip of outboard spiral wave to polar axis and point 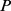 is very close to the origin of coordinate 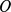. The distribution of contour is defined as follows
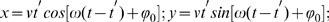
(5)


As shown in Fig. 5b, the intersection point for the outboard wavefront and the inboard wavefront is marked as point 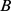, another monitoring point marked as 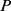 in Fig. 5a is used to detect the velocity for the wavefront of the outboard spiral wave toward to point 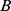. In our numerical studies, a tangent line, which is perpendicular to the polar axis, is plotted on the wavefront on the side closest to the inboard spiral wave and the point of tangency is marked as 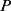. The motion of point 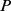 is measured by
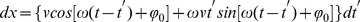


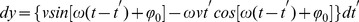
(6)


**Figure 6:**
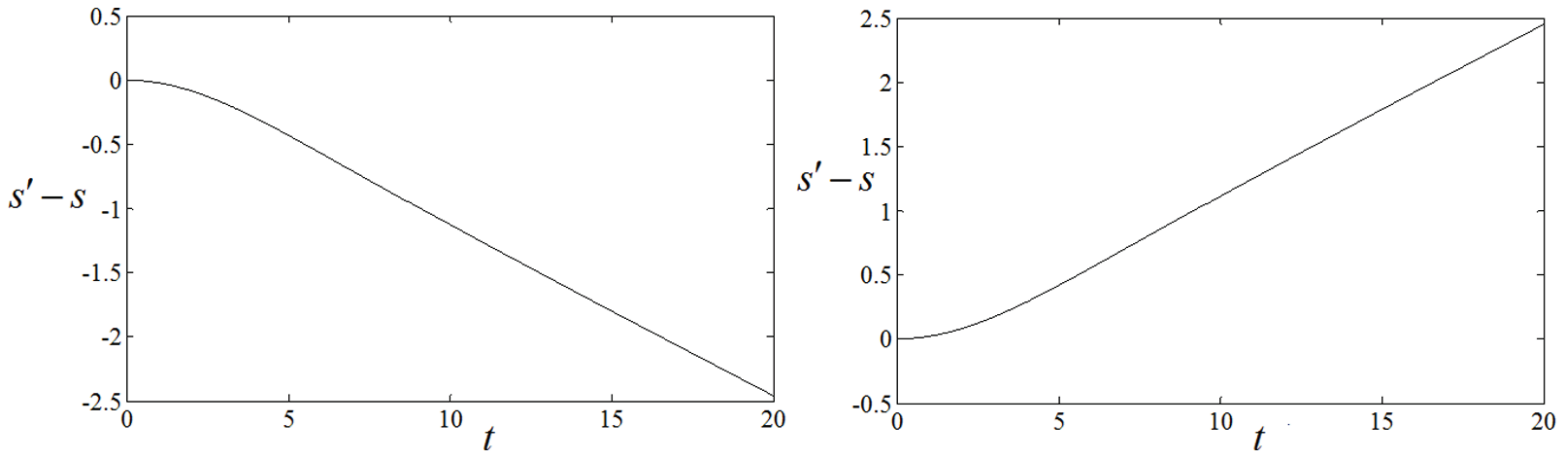
It depicts the time series (a) for 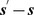 when coupling intensity for nodes outside of the inboard spiral wave is changed from 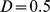 to 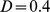. (b) It depicts the time series for 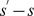 when coupling intensity for nodes inside of the outboard spiral wave is changed from 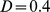 to 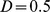. No external forcing current is imposed on the network(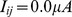). The vertical coordinate represents number (difference) of neurons that point 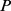 ever passed in certain transient period.

Clearly, the point 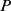 can be tracked according to the criterion as shown 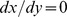 or 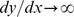. Based on the results as shown in Fig. 4, the double-spiral wave is stable(no expanding outside) and thus the intersection point 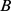 on the two adjacent wavefronts is fixed. Is supposes that the point 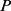 travels along the polar axis with a transient period 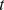 with a displacement as 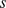, and a new displacement 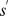 is calculated with the same transient period 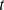 by decreasing the coupling intensity from 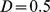 to 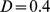 for those nodes outside of the inboard spiral wave. As a result, the inboard spiral wave expands to the border of the network and the point 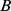 moves to the negative orientation of the polar axis for 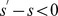; the outboard spiral wave is attracted by the inboard spiral wave and and the point 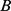 moves to the positive orientation of the polar axis for 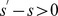; otherwise, the contour of the double-spiral wave keeps stable for 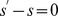. The value for 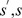 is calculated by recording the number of neurons that point 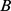 ever passed in a transient period 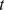. The numerical results confirm that the normal velocity and angular frequency for single-arm spiral wave is about 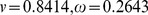 when the external forcing current for all nodes is selected 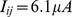, coupling intensity 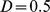 and parameter 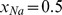. The normal velocity and angular frequency for single-arm spiral wave is about 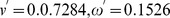 when the external forcing current for all nodes is changed to 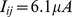, coupling intensity 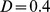 and parameter 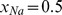. It is confirmed that the rotating spiral wave becomes slowly when the coupling intensity is decreased. In Fig. 6, the motion of point 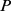 is detected by recording the time series for 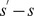 when the coupling intensity in the area for supporting the outboard spiral wave(or inboard spiral wave) is changed from 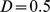 to 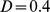.

**Figure 7:**
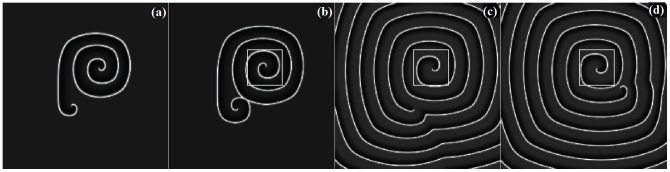
The development of the double-spiral wave is plotted with diversity in coupling intensity being considered. The coupling intensity for nodes outside the inboard spiral wave is decreased 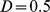 to 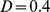 while the coupling intensity for the nodes in the inserted square(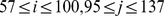) is kept as 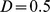. (a)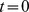, (b)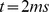, (c)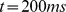, (d)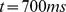, no external forcing current is imposed on the network(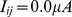).

The results in Fig. 6a confirm that the point 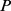 intends to move outwardly step by step when the coupling intensity for the nodes(corresponding to outboard spiral wave) outside of the inboard spiral wave is decreased from 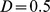 to 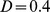. The results in Fig. 6b show that the point 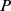 begins to be close to the inboard spiral wave slowly when the coupling intensity for nodes inside the outboard spiral wave is increased from 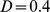 to 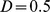. It indicates that the spiral wave could expand and propagate outwardly by decreasing the coupling intensity for nodes outside of the inboard spiral wave. Then the evolution of the spatiotemporal patterns is plotted in Fig. 7 to check the analysis as above.

**Figure 8:**
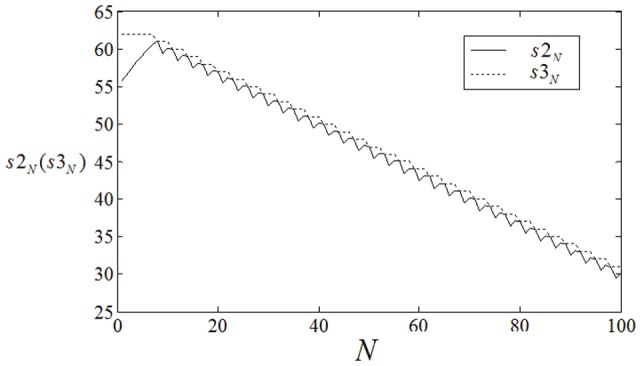
The development of 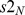,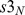 vs. collision times 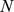 (a), the developed 2-arm spiral wave at 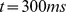. It begins from initial time 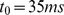, initial time 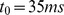, initial space 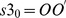 bridges 40 nodes in the network along polar axis. The coupling intensity for nodes (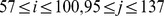) is selected as 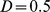, the other nodes are coupled with intensity 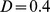, external forcing current on all nodes is selected as 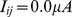.

**Figure 9:**
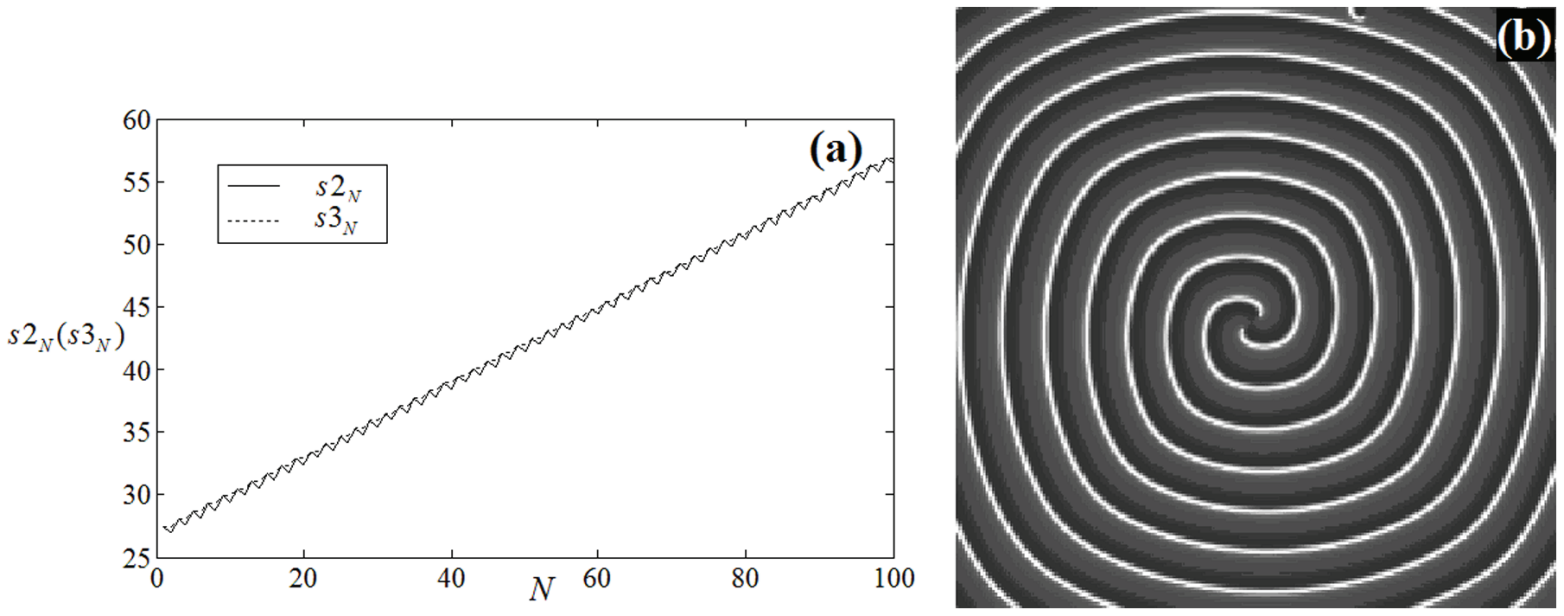
The development of 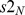,
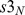
vs. collision times 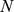 (a) when 2-arm spiral wave is induced in the center of the network. (b) The developed 2-arm spiral wave at 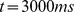. It begins from initial time 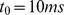, initial space 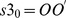 bridges 27 nodes in the network along polar axis. The coupling intensity for all nodes is selected as 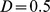, external forcing current on all nodes is selected as 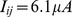.

**Figure 10:**
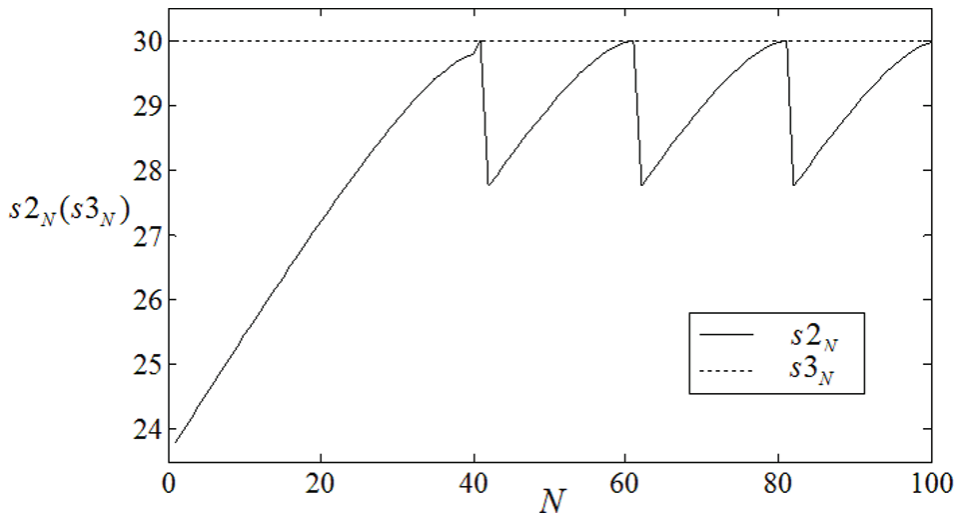
The development of 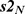,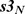 vs. collision times 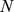 when 8-arm spiral wave is induced in the center of the network. It begins from initial time 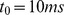, initial space 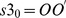 bridges 30 nodes in the network along polar axis. The coupling intensity for all nodes is selected as 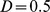, no external forcing current is imposed on the network(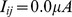).

**Figure 11:**
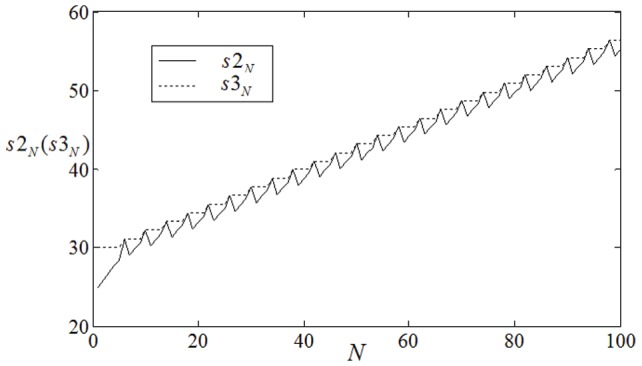
The development of 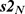,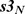 vs. collision times 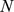 when 8-arm spiral wave is induced in the center of the network. It begins from initial time 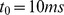, initial space 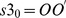 bridges 30 nodes in the network along polar axis. The coupling intensity for nodes (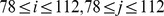) is selected as 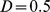, the other nodes are coupled with intensity 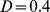, no external forcing current is imposed on the network(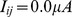).

The results in Fig. 7 confirm that the outboard spiral wave is repelled and the wavefront of the inboard spiral wave propagates outside to be closer to the tip of the outboard spiral wave in the network, a target-like wave is formed outside and the endpoint of the inboard spiral wave begins to curl due to a collision between the wavefront of inboard spiral wave and the tip of the outboard spiral wave, and thus a new double-spiral wave emerges in the network. In this way, this problem could be considered as that the tip of the outward spiral wave is unstable because a new double-spiral wave begins to emerge when the tip of outboard spiral wave collides with the wavefront of the inboard spiral wave. Therefore, it is better to use a fixed coordinate system to study this problem. The inboard spiral wave is often stable while the outboard spiral wave(disappears and emerges alternately) meanders in the network after a collision. Extensive numerical results confirm that inboard spiral wave(in the area with bigger coupling intensity) can not remove and drive the the outboard spiral wave to the border of network completely, however, the endpoint of the inboard spiral wave begins to curl and form a new double-spiral wave when the tip of the last outboard spiral wave collides with the outwarding wavefront of the inboard spiral wave. To solve this problem, the origin of coordinates is fixed on the center of the inboard spiral wave, and the connection line between the center of outboard and inboard spiral wave is used as polar axis. The monitoring point 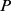 is moving but could be detected as above, and thus the displacement 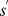
or 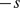 is replaced by a new segmented function 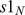 because the 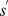 or 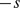 is changeable after each collision. The position for point 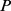 at fixed time 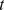 could be calculated according to the Eq.(7) as follow
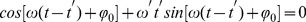
(7)

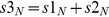
(8)

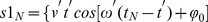
(9)

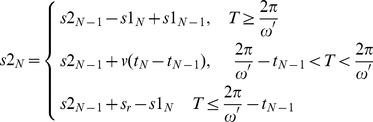
(10)Where 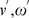 is the normal velocity and angular frequency of the outboard spiral wave, 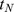 is time when the outboard spiral wave collides with the inboard spiral wave for 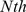 times, 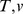 is the rotation period and normal velocity for the inboard spiral wave, 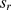 is the displacement along polar coordinate after a meandering of the outboard spiral wave after each collision for wavefronts, 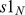 (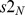) denotes distance between the intersection point 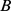 to the center of the outboard spiral wave(inboard spiral wave) when the wavefronts collide. In the numerical studies, initial time 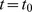 and initial distance 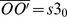 are fixed, then 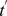 is detected according to Eq.(7), and 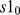, 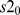 could be calculated according Eq.(9), Eq.(10), respectively. The collision position 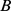 is approached by detecting the position 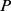 at each collision time of wavefronts, repeating the iterative operation according to Eq.(7) to Eq.(10), and the position 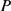 could by detected by calculating the values of 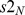. The results are plotted in Fig. 8, Fig. 9, Fig. 10, and Fig. 11 under different conditions.

The results in Fig. 8 show that the position 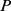 (or collision point 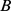)becomes closer to 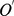 that the inboard spiral fails to enlarge its area. In this way, a single double spiral wave cannot grow up to occupy the network completely. Furthermore, we investigate the case when two double-spiral waves are initiated in the network as initial values, and the results are shown in Fig. 9.

The results in Fig. 9 confirm that the collision point 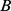 keeps away from the point 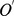 step by step, it indicates that the two double-spiral waves develop to form a 2-arm spiral wave and occupy the network completely within certain transient period. Extensive numerical results confirm that a 2-arm spiral wave exists in a local area of the network but fails to grow up completely when the external forcing current 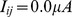 is used in the network. Then we calculated the normal velocity and angular frequency for a single-arm spiral wave and 2-arm spiral wave at 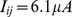 and 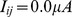, respectively. The normal velocity is 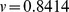 for a single-arm spiral wave and 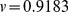 for a 2-arm spiral wave when all nodes are imposed forcing current 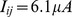, while the normal velocity is 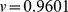 for a single-arm spiral wave and 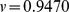 for a 2-arm spiral wave when all nodes are imposed forcing current 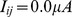. Clearly, the normal velocity for 2-arm spiral wave becomes smaller compared with a corresponding single-arm spiral wave when no external forcing current is imposed on the network(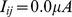), while the normal velocity for 2-arm spiral wave becomes bigger than a single-arm spiral wave when the external forcing current is increased to 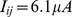. Extensive numerical results confirm that a 2-arm spiral wave still be developed to occupy the network completely by introducing diversity in coupling intensity in the network under 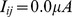. It is more difficult to generate multiarmed spiral wave when the arm number is high, for simplicity, the case for 8-arm spiral wave is investigated at 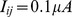, 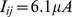, respectively.

**Figure 12:**
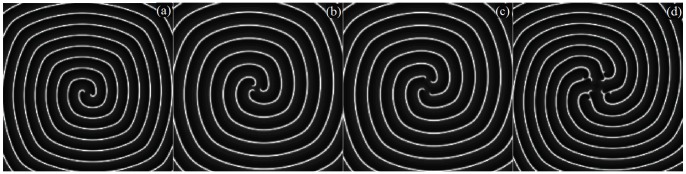
Multiarmed spiral waves with different arm numbers are developed in the network with certain transient period. For(a) 2-arm spiral wave at 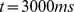, for (b)3-arm spiral wave 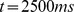, for (c)4-arm spiral wave at 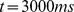, for (d)8-arm spiral wave at 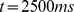, no external forcing current is imposed on the network. The nodes close to the tips of inboard spiral waves are coupled with intensity 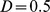, other nodes are coupled with intensity 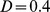
(similar to the illustration in Fig. 7).

The results show that that the collision point 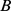 cannot keeps away from the point 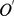 monotonously but does oscillate in a periodical way when the external forcing current is selected as 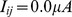, it indicates that the 8-arm spiral wave just emerges in a local area and could not grow up to occupy the network completely. Then it introduces diversity in coupling intensity into the network, and it is found that multiarmed spiral wave could be developed to occupy the network completely in certain transient period. In the numerical studies, the coupling intensity for nodes in the area close to the inboard tip of multiarmed spiral wave is selected by 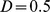 and the rest nodes are coupled with intensity 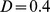
(similar to Fig. 7), the results are shown in Fig. 11, Fig. 12.

The results in Fig. 11 confirm that the collision point 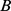 begins to leave the point 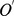 gradually when different coupling intensities are introduced into the network even if no external forcing current is imposed on the network, then the developed multiarmed spiral waves with different arm numbers are plotted in Fig. 12.

The snapshots in Fig. 12 show that multiarmed spiral wave could be induced and these waves rotate stably in the network with certain transient period.

In a summary, multiarmed spiral waves could be induced by introducing spatial forcing currents with certain transient period into the network followed by an appropriate shift in conductance in Sodium(via ion channels blocking). A group of double-spiral waves are generated when spatial forcing currents are imposed on several controlled nodes with symmetrical distribution in the network followed a shift in conductance, and these double-spiral waves could form multiarmed spiral wave with different arm numbers in a local area. It is found that these local multiarmed spiral waves can grow up to occupy the network completely when diversity in coupling intensity is introduced into the network even if no forcing current is imposed on the network. Similar to the illustration as shown in Fig. 3a, a group of controlled nodes are distributed in the network symmetrically, double-spiral waves could be induced when spatial forcing currents are imposed on the three controlled nodes(three nodes should be controlled at least so that a double spiral could be approached by a short polygonal line). In fact, these double-spiral waves are often unstable except for those double-spiral waves rotate in the same direction synchronously. The external spatial forcing currents on the network generate target-like waves, a shift of conductance in sodium cause breakup of a group of target-like waves and spiral segments is formed. Multiarmed spiral wave could be induced in a local area due to frequent collision between double-spiral waves developed from spiral segments. We also check this problem by changing the conductance in Potassium, and it is found some multiarmed spiral wave could be induced even though these muiltiarmed spiral waves are also unstable in the network completely. Finally, it is important to discuss the biological relevance of our findings. A survival of multiarmed spiral wave is much dependent on the media with low excitability while its propagation in the media prefers to high excitability, thus it indicates that multiarmed spiral wave is seldom observed in experiments and numerical studies. Multiarmed spiral wave is also self-sustained, and its stability is helpful to conserve the ordered state of the media. The scheme of spatial current forcing with diversity could be a good application instance of deep brain stimulation, which is often used to treat patients with Parkinson's disease [67]. In an experimental way, a few of electrodes with constant forcing current are imposed on the media symmetrically, a target wave emerges close to each electrode due to local pacing and gradient effect, spiral waves could be induced in the media after collision and cooperation between these target waves, thus the media could keep ordered state under a pacemaker generated by the spiral wave even if the electrodes are removed.

## Conclusions

The selection of multiarmed spiral wave with different arm numbers in the regular network of Hodgkin-Huxley neurons is detected and discussed. Spatial forcing currents with diversity are imposed on neurons to form target-like wave, the diversity in forcing currents is removed and the conductance is changed to break the non-persistent target wave to develop multiarmed spiral waves. It is found that multiarmed spiral wave with bigger number of arm often is unstable and fails to occupy the network completely. However, multiarmed spiral wave with bigger number of arms can be developed to occupy the network completely by introducing diversity in coupling intensity in the network. In fact, a double-spiral wave is induced when external forcing currents with diversity are imposed on three adjacent nodes(not in a line) followed by a change in bifurcation parameter such as conductance, a double-spiral wave is developed from the broken spiral segments and a group of double-spiral waves could rotate in the same direction to form some multiarmed spiral waves in the network. Our numerical results confirm that multiarmed spiral wave with different arm numbers such as *l* = 2, 3, 4,.. could be developed in the network. More interesting, it is confirmed that higher excitability for neurons in the network is useful for the developed multiarmed spiral wave to propagate outside while it is destructive to from multiarmed spiral wave in the transient period, as a result, it gives some clues to understand that multiarmed spiral waves are often unstable and infrequent in most of the biological systems.

## Supporting Information

Movie S1 **Supporting flash for 8-arm spiral wave.**
(SWF)

Movie S2 **Supporting flash for random multi-armed spiral wave.** The two short movies are supplied to observe the formation of multiarmed spiral waves in the network.
(SWF)
